# Peripheral Vagus Nerve Stimulation Significantly Affects Lipid Composition and Protein Secondary Structure Within Dopamine-Related Brain Regions in Rats

**DOI:** 10.1007/s12017-015-8349-7

**Published:** 2015-04-19

**Authors:** Artur Dawid Surowka, Anna Krygowska-Wajs, Agata Ziomber, Piotr Thor, Adrian Andrzej Chrobak, Magdalena Szczerbowska-Boruchowska

**Affiliations:** AGH University of Science and Technology, Faculty of Physics and Applied Computer Science, Al. Mickiewicza 30, 30-059 Kraków, Poland; Department of Neurology, Jagiellonian University Medical College, Kraków, Poland; Department of Pathophysiology, Jagiellonian University Medical College, Kraków, Poland; Jagiellonian University Medical College, Kraków, Poland

**Keywords:** Vagus nerve, Electrical stimulation, Gastrointestinal system, Dopaminergic system, Fourier transform infrared microspectroscopy

## Abstract

Recent immunohistochemical studies point to the dorsal motor nucleus of the vagus nerve as the point of departure of initial changes which are related to the gradual pathological developments in the dopaminergic system. In the light of current investigations, it is likely that biochemical changes within the peripheral nervous system may influence the physiology of the dopaminergic system, suggesting a putative role for it in the development of neurodegenerative disorders. By using Fourier transform infrared microspectroscopy, coupled with statistical analysis, we examined the effect of chronic, unilateral electrical vagus nerve stimulation on changes in lipid composition and in protein secondary structure within dopamine-related brain structures in rats. It was found that the chronic vagal nerve stimulation strongly affects the chain length of fatty acids within the ventral tegmental area, nucleus accumbens, substantia nigra, striatum, dorsal motor nucleus of vagus and the motor cortex. In particular, the level of lipid unsaturation was found significantly increasing in the ventral tegmental area, substantia nigra and motor cortex as a result of vagal nerve stimulation. When it comes to changes in protein secondary structure, we could see that the mesolimbic, mesocortical and nigrostriatal dopaminergic pathways are particularly affected by vagus nerve stimulation. This is due to the co-occurrence of statistically significant changes in the content of non-ordered structure components, alpha helices, beta sheets, and the total area of Amide I. Macromolecular changes caused by peripheral vagus nerve stimulation may highlight a potential connection between the gastrointestinal system and the central nervous system in rat during the development of neurodegenerative disorders.

## Introduction

Parkinson’s disease (PD) is a progressive neurodegenerative process that takes years to fully develop. The histopathological features of PD include the loss of dopamine in nigrostriatal neurons as well as the presence of α-synuclein-positive, intracytoplasmic inclusions known as Lewy bodies both in the dopaminergic cells of the substantia nigra (SN) and in other regions of the central nervous system (Dickson 2012). This neurodegenerative disorder is characterized clinically by postural instability, bradykinesia, rigidity and tremor (de Lau and Breteler 2006). However, recent studies also indicate that non-motor symptoms such as depression and gastrointestinal disturbance may precede motor signs in PD (Abbott et al. 2001; Wolfgang 2010). It has also been suggested that affective symptoms may occur as initial symptoms of PD many years before motor signs (Fukunishi et al. 1991;Guze and Barrio 1991; Kostić et al. 1996; Mellers et al. 1995). Immunohistochemical studies point to the dorsal motor nucleus of the vagus nerve (DMV) as the point of departure for initial changes related to the gradual development of pathologies in the human dopaminergic system, including both PD and depression (Braak et al. 2003). Recently, it has been demonstrated that DMV is a part of the dorsal motor complex, composed the nucleus tractus solitarii (NTS) and area postrema (Wang et al. 2014). NTS projects to many brain areas including the hindbrain, midbrain and forebrain, and dopamine-related brain structures—the ventral tegmental area (VTA) and nucleus accumbens (NAC) (Merchenthaler et al. 1999; Rinaman 2010). In addition, the vagus nerve connects the DMV with, among others, the gastrointestinal system, which is considered to be significantly affected at the onset of PD, even before motor signs occur (Braak et al. 2003). This suggests, therefore, some putative action of the peripheral nervous system during the progressive pathology of the human dopaminergic system. However, apart from many general neurobiological studies (Braak et al. 2003; Greene 2014), there is no current knowledge of the exact molecular mechanisms that underlie the effect of vagal nerve activity on the functioning of the central monoaminergic system. Thus, the question arises whether the activity of the vagal nerve might have any potential biochemical effects (either beneficial or destructive) in various dopamine-related regions of the brain.

Much work has been done to explore the variety of pathological mechanisms that underlie the pathophysiology of PD. Roles has been suggested for oxidative stress neurotoxicity, mitochondrial dysfunctions, excitotoxicity, acute/subacute inflammatory responses, and changes in either protein secondary structure or metabolism (Ronald et al. 2012). Although there are still many questions to answer, the role of the initial changes which are associated with the development of pathologies in the human dopaminergic system has recently grown in importance. Taking into account the fact that the dorsal motor nucleus of the vagal nerve can be considered as the point of departure of early changes related to the gradual development of pathologies in the dopaminergic system, we investigated the influence of vagus nerve stimulation on the biochemical composition of dopamine system in the rat brain. In particular, we examined the effect of chronic, unilateral electrical vagus nerve stimulation on changes in lipid composition and in protein secondary structure within dopamine-related brain structures in rats.

In line with our previous studies, we have shown that the chronic stimulation of the vagal nerve (CVNS) affected both the level of selected neurometabolites (3-methoxytyramine, 3,4-dihydroxyphenylacetic acid, 5-hydroxyindoleacetic acid) and trace elements (Fe, Zn, Cu, Ca) within various parts of the dopaminergic system of rats (Ziomber et al. 2012; Szczerbowska-Boruchowska et al. 2012). We also found that chronic stimulation of the vagus nerve function leads to the inhibition of dopamine but not the serotonin neurons in rat brain structures (Ziomber et al. 2012*)*. Nevertheless, in order to compare these results with the real-world pathophysiology of the early stages of PD, it is crucial to assess whether chronic stimulation of vagus nerve also affects the main organic biomolecules such as proteins and lipids within dopamine-related brain structures. As pathomechanisms related to PD may affect the main biomolecular components of brain tissue structures, we studied lipid and protein changes within the dopamine-related brain structures of rats following chronic vagal nerve stimulation. We also aimed at a determination of the similarities/dissimilarities in lipid and protein changes occurring dopaminergic neural pathways. For this purpose, we used Fourier transform infrared microspectroscopy (FTIRMS), coupled with statistical and chemometric analyses. FTIRMS couples IR spectroscopy with microscopy to determine the chemical composition of specimens on a microscopic scale, including both the content of biological macromolecule functional groups as well as the secondary structure of proteins (Stuart 2004). This is the first study to our knowledge which uses infrared microspectroscopy to investigate biomolecular changes within dopamine-related brain structures upon peripheral vagus nerve stimulation.

## Materials and Methods

### A Procedure for Microchip Electrical Stimulation of Vagus Nerve

Research was carried out on 12 male Wistar rats. All the animals were subjected to surgical implantation of a microchip in the abdominal region of the left vagus nerve. The animals were divided into two groups. The first one (MC, *n* = 6) underwent microchip stimulation of the left vagus nerve, while the control group (C, *n* = 6) was subjected to sham laparotomy.

For the purpose of surgical laparotomy, the subdiaphragmatic part of the left vagus nerve was connected to a 1-cm-diameter silicon-coated (RTV 3140, Dow Corning), battery-driven microchip. Experiments were carried out after 12 h of food deprivation, an empty stomach making it easier to access the nerve following the administration of pentobarbital (vetbutal, dose: 25 mg/kg of body mass; Biowet, Puławy, Poland) intraperitoneally. Once the abdominal part of the left vagus nerve was localized and electrically connected and the wounds were closed, the rats were moved to cages and subjected to 7 days of stimulation. Food and water were allowed ad libitum during the whole experiment. The period of the stimulating signal was 20 s, its duration was 0.1 s, and the amplitude was 200 mV. Finally, following electric stimulation, all the rats were guillotined. All procedures involving animals were approved by the Jagiellonian University Bioethical Committee (20/11/2009).

### Sample Preparation

**Fig. 1:**
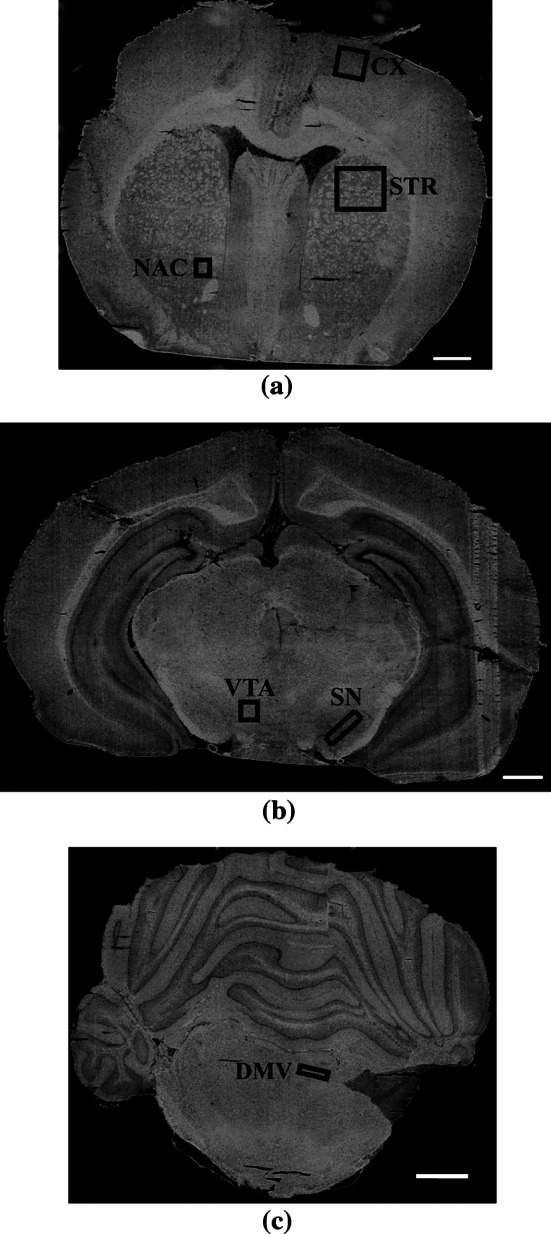
Visual images of unstained sections of rat brain showing the loci of the areas analyzed using the FTIRMS technique; **a** CX—motor cortex, STR—striatum, the medial part of caudate putamen; NAC—nucleus accumbens; **b** VTA—ventral tegmental area, SN—substantia nigra; **c** DMV—dorsal motor nucleus of the vagus nerve. *Scale bars* 1 mm

Immediately after decapitation, the brain of each animal was rapidly removed and preserved in liquid nitrogen. Before measurements were taken, the specimens were carefully cut into 10-μm-thick slices at −16 °C, mounted onto Low e-MirrIR Microscope Slides (Kevely Technologies) and subsequently freeze-dried at −80 °C. For this study, coronal sections containing the VTA, NAC, motor cortex (CX), SN and striatum (STR) (including fiber bundles (STR_F) and corpus striatum spaces (STR_E)), and the DMV were used to analyze the effects of vagus nerve stimulation. Images of the coronal sections showing the location of these brain areas are shown in Fig. 1.

### FTIRMS Spectra Acquisition

Spectral images were collected in transflection mode using an infrared microscope (Continuum—Thermo Scientific), coupled with an FTIRMS spectrometer (Nicolet 8700—Thermo scientific), equipped with a liquid N_2_-cooled mercury cadmium telluride detector. All acquisitions were carried out in air, in the mid-IR spectral range (4000–720 cm^−1^), with a spectral resolution of 8 cm^−1^, by means of an 18 × 18 (μm × μm) aperture. Stage control and data collection were performed by means of OMNIC 8.0 software (Thermo-Nicolet, Madison, Wisconsin).

### Data Preprocessing

**Fig. 2:**
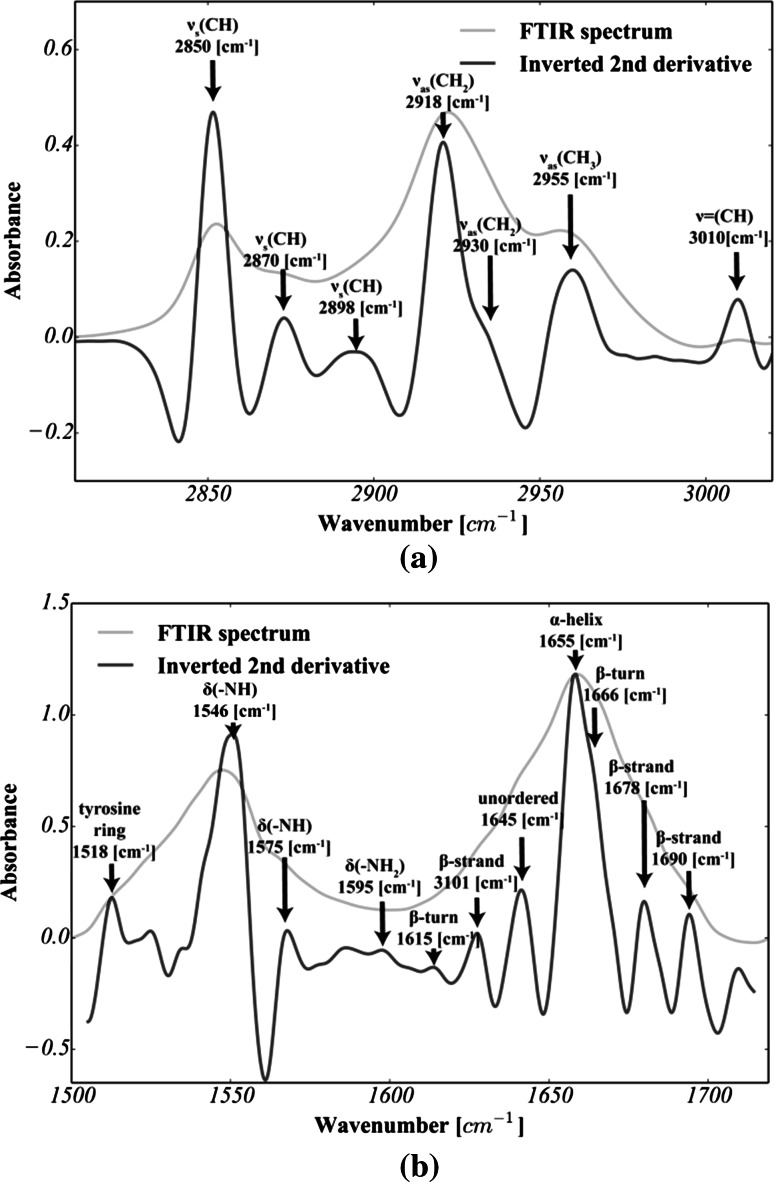
Second-derivative FTIRMS spectra of: **a** lipid massive; **b** Amide I–II spectral range (seven-point Savitzky–Golay filtration was applied to produce decent-looking spectra). For the sake of clarity, the second derivative was multiplied by −1

For each scanned area, at least 30 spectra were chosen from the FTIRMS images (see Fig. 2a, b). Analysis of the data was carried out using a home-made code written in Python with NumPY (van der Walt et al. 2011*)*, SciPY (van der Walt et al. 2011*)*, lmfit (http://lmfit.github.io/lmfit-py), and matplotlib (Hunter 2007) packages. First, each spectrum was cut to 3050–2800 cm^−1^ (lipids) and 1800–1500 cm^−1^ (proteins) spectral intervals, baseline corrected (polynomial baseline) and vector normalized. Following data preprocessing, all FTIRMS spectra were subjected to curve fitting and chemometric procedures, as described below.

### Curve Fitting and Statistical Data Analysis

**Fig. 3:**
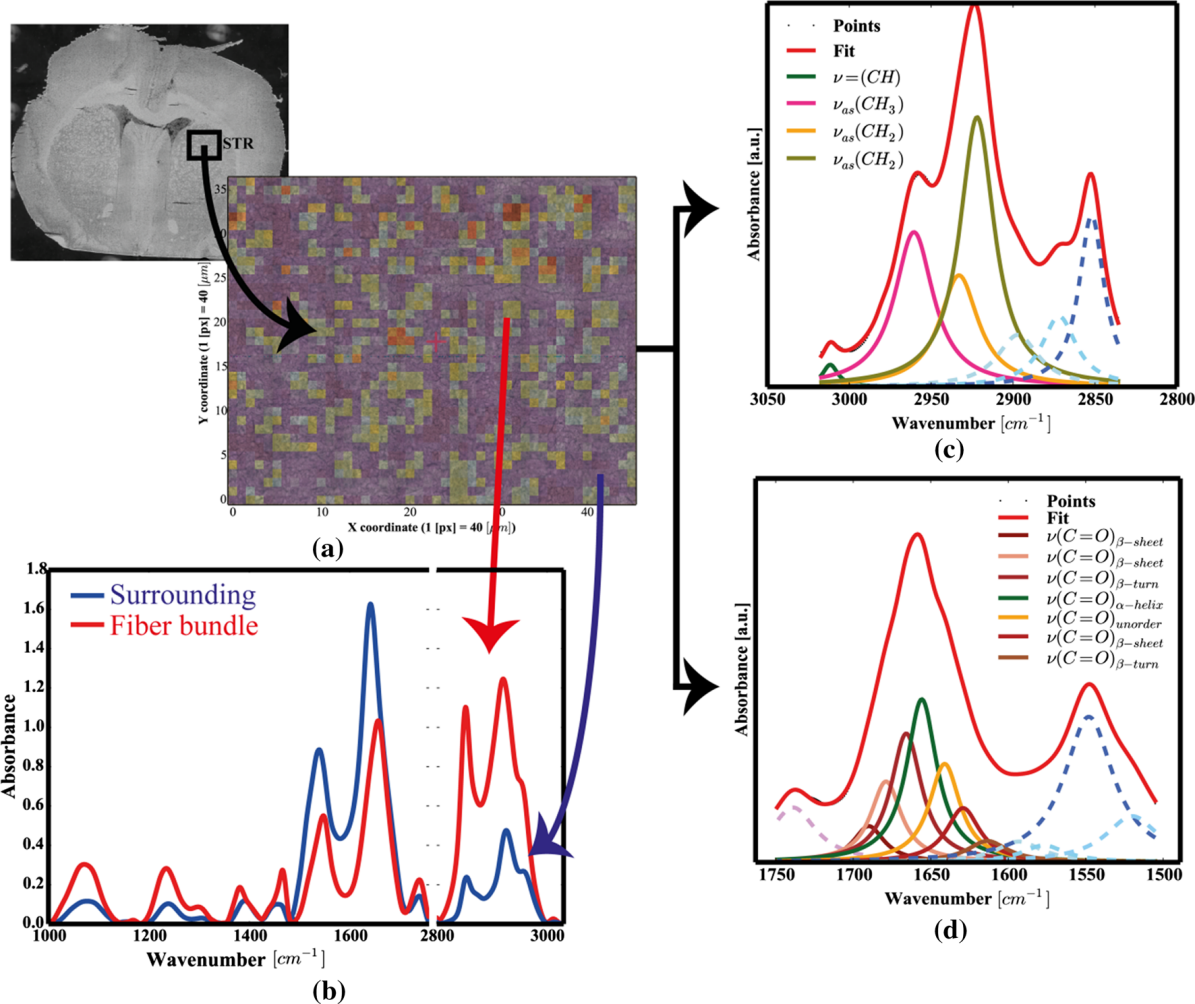
**a** FTIRMS map of STR; **b** typical FTIRMS spectrum of STR, extracted from FTIRMS map; *curve* fitting of the FTIRMS spectrum for: **c** lipid massive; **d** Amide I

To determine whether any changes in lipid content and in protein secondary structure occur, all spectra were analyzed by means of curve fitting approach. The home-made code written in Python, using the standard Lorentzian peak fitting model, was applied as suggested by Gough et al. 2010. The fatty acyl absorption range and Amide I and II bands were analyzed separately. First, the initial position of each band was established based on the second-derivative spectra (see Fig. 2) and assigned as suggested by Petibois and Déléris (2006), Kolta et al. (1999) and Gough et al. (2010). Thus, as shown in Fig. 3c, for lipid groups, seven bands were fixed at 3012, 2951, 2930, 2018, 2899, 2870 and 2851 cm^−1^, and as shown in Fig. 2d, seven bands were fixed at 1691, 1680, 1669, 1654, 1645, 1625 and 1612 cm^−1^ for protein groups.

In order to minimize the root-mean-square difference between the model and experimental data, all fitting procedures involved an iterative Levenberg–Marquardt algorithm with a maximum of 500 iterations. Once the band positions were established, the heights of all peaks were initially set to a half of the maximum value within the analyzed range, while the start values of the standard deviation (SD) parameter were set to 12 cm^−1^. An acceptable fit was generated by the calculation of all parameters (position, height and SD) using reasonable parameter bounds (position ±2 cm^−1^; height from 1 to 100 % of the maximum value within the analyzed range; SD: ±2 cm^−1^; *R*^2^ > 0.95). Finally, a set of over 5500 spectra was analyzed, while the net peak areas of selected bands were subjected to further statistical analysis (Petibois and Déléris 2006; Wehbe et al. 2010).

On completion of the curve fitting procedure, the statistical analysis of biomolecular changes within the dopaminergic system of the animals was carried out. For this purpose, the level of lipid unsaturation (UNSAT) and the fatty acyl chain length (FACL) were calculated according to the following formulas (Petibois and Déléris 2006; Leskovjan et al. 2010):

1.1 $${\text{UNSAT = }}\frac{{\nu = \left( {\text{CH}} \right)}}{{\nu_{\text{as}} \left( {{\text{CH}}_{3} } \right)}}$$

1.2 $${\text{FACL = }}\frac{{\nu_{\text{as}} \left( {{\text{CH}}_{ 2} } \right)}}{{\nu_{\text{as}} \left( {{\text{CH}}_{ 3} } \right)}}$$

where $\nu = \left( {\text{CH}} \right)$ denotes the net peak area of band at 3012 cm^−1^; $\nu_{\text{as}} \left( {{\text{CH}}_{ 2} } \right)$ the net peak area of bands at 2930 and 2918 cm^−1^; and $\nu_{\text{as}} \left( {{\text{CH}}_{ 3} } \right)$ the net peak area of band at 2958 cm^−1^.

The total content of lipid within analyzed areas was calculated as a net sum of all vibration modes within 3012–2800 spectral range:

1.3 $$TLIP = \sum\limits_{i} \nu ({{\text{CH}}_{ 2} })_i + \sum\limits_{j} {\nu ({{\text{CH}}_{ 3} })_j }$$

where *i, j* = indexes attributed to different vibration modes assigned to both symmetric and asymmetric modes of vibration for ${\text{CH}}_{ 2}$ and ${\text{CH}}_{ 3}$ groups, respectively.

In turn, to establish whether any changes in protein secondary structure contribute to the effects of CVNS, the total area of Amide I (TPRO) as well as the percentage content of alpha helices (%ALPHA), random coils (%UNORDER), beta sheets (%BETA_S) and beta turns (%BETA_T) was evaluated using Eqs. (2.1–2.5) as follows (Petibois and Déléris 2006):

2.1 $$TPRO = \nu ({\text{C = O)}}_{{\alpha - {\text{helix}}}} + \nu ({\text{C = O}})_{\text{unorder}}+ \nu ({\text{C = O)}}_{{\beta - {\text{sheet}}}} + \nu ({\text{C = O)}}_{{\beta - {\text{turn}}}}$$

2.2 $$\% {\text{ALPHA = }}\frac{{\nu \left( {\text{C = O}} \right)_{{\alpha - {\text{helix}}}} }}{\text{TPRO}} \times 1 0 0\%$$

2.3 $$\% {\text{UNORDER = }}\frac{{\nu \left( {\text{C = O}} \right)_{\text{unorder}} }}{\text{TPRO}} \times 1 0 0\%$$

2.4 $$\% {\text{BETA}}\_{\text{S}} = \frac{{\nu \left( {{\text{C}} = {\text{O}}} \right)_{{\beta - {\text{sheet}}}} }}{\text{TPRO}} \times 100\%$$

2.5 $$\% {\text{BETA}}\_{\text{T = }}\frac{{\nu \left( {\text{C = O}} \right)_{{\beta - {\text{turn}}}} }}{\text{TPRO}} \times 1 0 0\%$$

where $\nu \left( {\text{C = O}} \right)_{{\alpha - {\text{helix}}}}$ denotes the net peak area of band at 1654 cm^−1^; $\nu \left( {\text{C = O}} \right)_{\text{unorder}}$ the net peak area of band at 1645 cm^−1^; $\nu \left( {\text{C = O}} \right)_{{\beta - {\text{sheet}}}}$ the net peak area of bands at 1691, 1680 and 1625 cm^−1^; and $\nu \left( {\text{C = O}} \right)_{{\beta - {\text{turn}}}}$ the net peak area of bands at 1668 and 1615 cm^−1^.

**Fig. 4:**
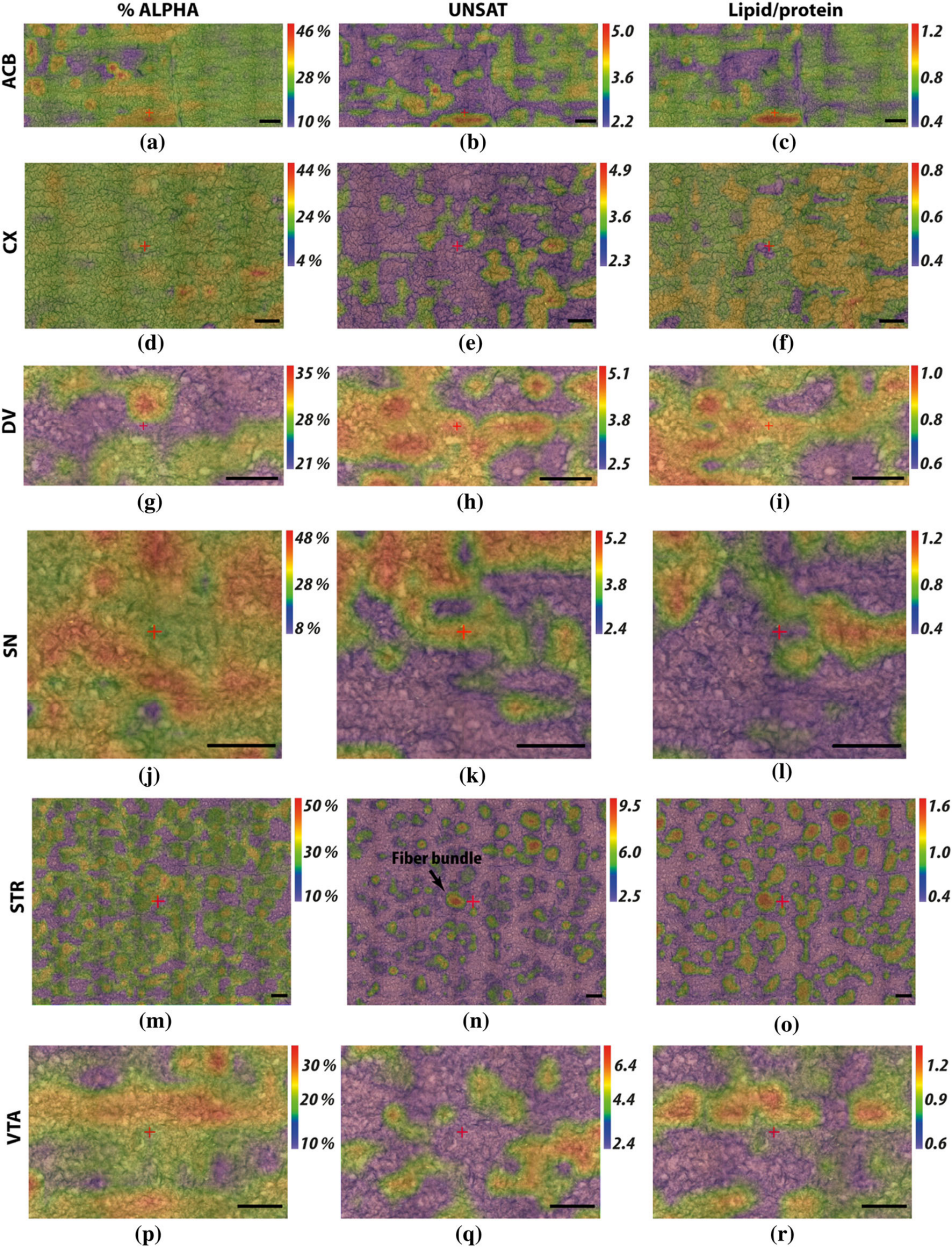
Exemplar FTIRMS images presenting 2D distribution of selected parameters within brain regions analyzed for a stimulated animal;  %ALPHA according to Eq. 2.2; UNSAT according to Eq. 1.1; lipid/protein according to Eq. 1.3

With numerical values calculated as above, two-dimensional distributions for  %ALPHA, UNSAT and TLIP are shown in Fig. 4 for each anatomical structure analyzed.

Finally, based on the numerical values calculated above, the final statistical quantification relied upon comparisons between the MC and C groups of animals. Since the Shapiro–Wilk test for normality (at *p* = 0.05 level) did not reveal appropriate agreement with a normal distribution for all the parameters, a nonparametric Mann–Whitney *U* test was applied. Thus, for each part of the rat brain, the median values of the analyzed parameters were compared using a nonparametric Mann–Whitney *U* test both for the MC and C groups of animals, while all *p* values of less than 0.05 were considered to exhibit statistical significance. As the left branch of the vagus nerve was subjected to electric stimulation, the left (L) and right hemispheres (R) were analyzed separately.

## Results

### Curve Fitting

In order to study the biomolecular changes induced by CVNS, FTIRMS coupled with the curve fitting approach was exploited. In the literature, analogical approaches have been extensively used (Surowka et al. 2014*)*. Particularly, spectral area integrations of the 3012, 2951 and 2921 cm^−1^ absorption bands were used to analyze the changes in lipid composition of brain tissue sections for animal models of neurological diseases (Petibois and Déléris 2006; Wehbe et al. 2010). As reported, the curve fitting approach can provide an insight into the overlapped Amide I modes of vibrations (Stuart 2004). These bands are, therefore, attributed to various protein conformations as follows: alpha helix (1654 cm^−1^), beta sheets (1691, 1680 and 1625 cm^−1^), beta turns (1669 and 1612 cm^−1^) and random coils (1645 cm^−1^). In this work, we coupled a similar approach with statistical analysis to compare the lipid composition and the protein secondary structure of tissue sections belonging to electrically stimulated and control animals. In order to represent the mean values and the distribution of the data, traditional box plots were used (see Figs. 3, 4). Red boxes apply to those stimulated and green lines to the control group of animals (cf. Figs. 3, 4).

**Fig. 5:**
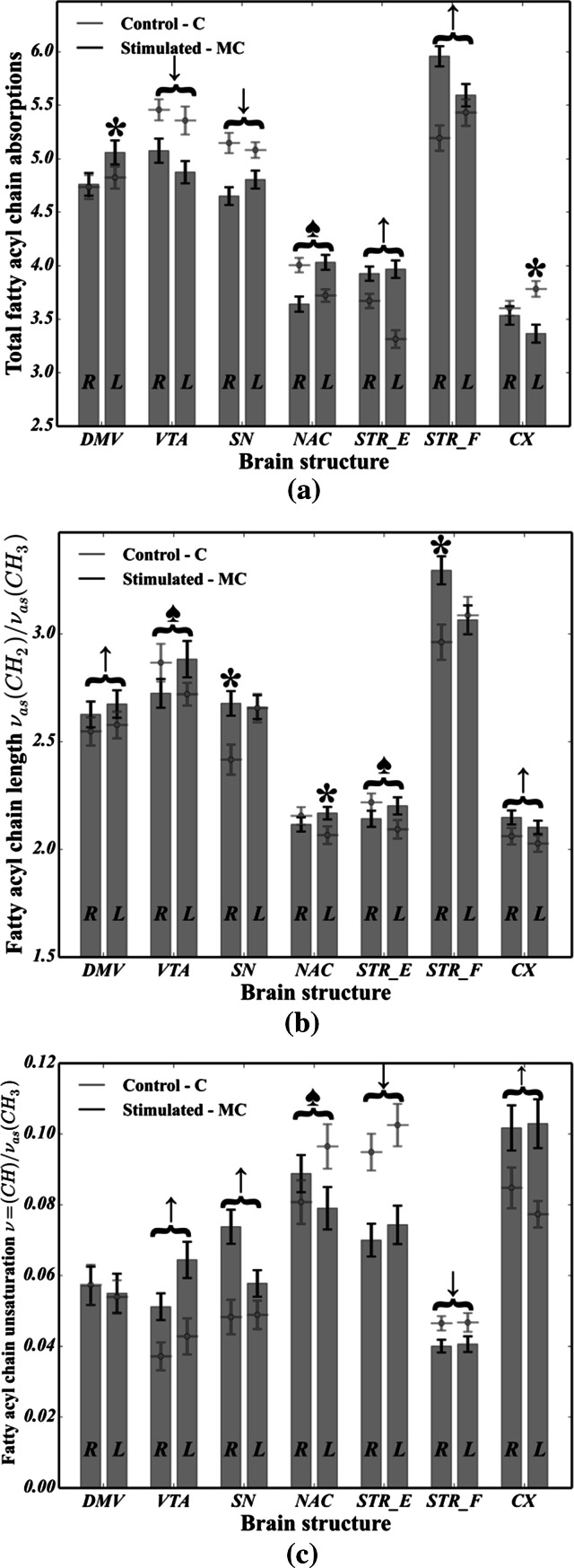
Comparison of changes in lipid composition between the microchip-stimulated group (MC) and the control (C): **a** total fatty acyl chain absorptions; **b** the FACL; **c** the level of lipid unsaturation. R—right hemisphere; L—left hemisphere. Assignments: *upward arrow* a statistically significant (*p* < 0.05) increase was observed in both hemispheres; *downward arrows* a statistically significant (*p* < 0.05) decrease was observed in both hemispheres; *black spade suit* asymmetric change (*p* < 0.05) occurred in both hemispheres (i.e., a decrease in the first one and an increase in the second one); *asterisk* a statistically significant change (*p* < 0.05) in a specific parameter was observed in one hemisphere

In the main, as shown in Fig. 5, it is apparent that in the majority of brain areas analyzed, many statistically significant differences in the total fatty acyl chain absorptions (cf. Figure 5a), lipid unsaturation (cf. Fig. 5b) and FACLs (cf. Fig. 5c) were noted. From the data in Fig. 5a, it can be seen that the total fatty acyl chain absorptions decreased for the MC group in VTA, SN as well as in the right hemisphere for NAC and in the left hemisphere for CX. It should, however, be noted that for STR_E, STR_F and NAC (of the left hemisphere), a noticeable (*p* < 0.05) increase occurred for stimulated rats. In particular, as regards lipid saturation (see Fig. 5b), all but three of all pairwise comparisons between MC and C cases were highly significant (*p* ≪ 0.05), indicating that CVNS strongly affects the fatty acid chain length within VTA, SN, NAC, STR_F, DMV, STR_E and CX. The results presented in Fig. 3c indicate that the level of lipid unsaturation increased for the MC group in VTA (*p* < 0.05 for both hemispheres), SN, CX, while for both STR_F and STR_E (*p* < 0.05 for both hemispheres) and for NAC (*p* < 0.05 of left hemisphere), it markedly decreased for stimulated rats compared with control animals.

**Fig. 6:**
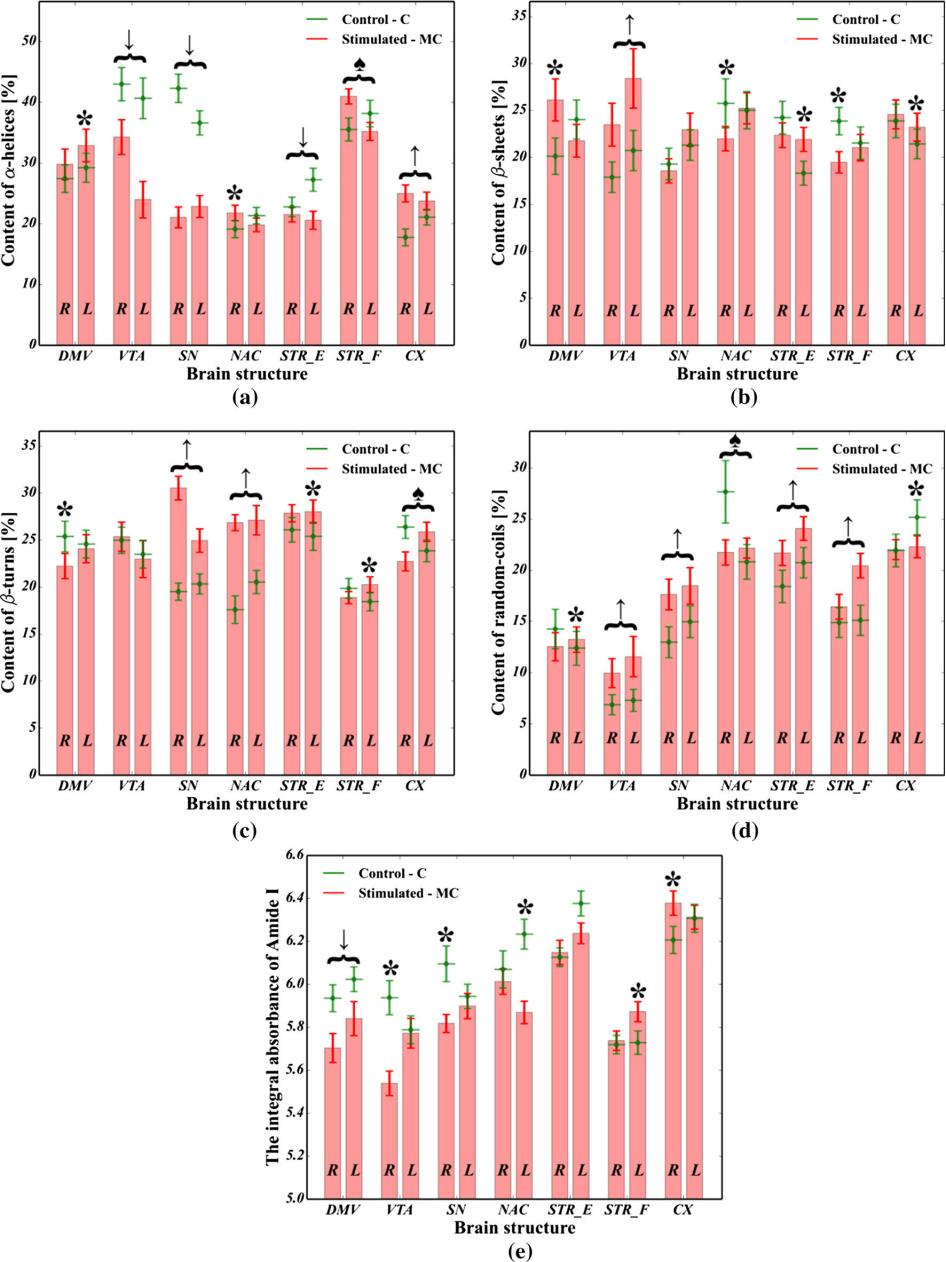
Comparison of changes in protein composition between the microchip-stimulated group (MC) and the control (C): **a** the percentage content of alpha helices; **b** percentage content of beta sheets; **c** percentage content of beta turns; percentage content of random coils; **e** integrated area of Amide I. R—right hemisphere; L—left hemisphere. Assignments: *upward arrow* a statistically significant (*p* < 0.05) increase was observed in both hemispheres; *downward arrow* a statistically significant (*p* < 0.05) decrease was observed in both hemispheres; *black spade suit* asymmetric change (*p* < 0.05) occurred in both hemispheres (i.e., a decrease in the first one and an increase in the second one); *asterisk* a statistically significant change (*p* < 0.05) in a specific parameter was observed in one hemisphere

The results obtained from the analysis of the protein secondary structure are shown in Fig. 6. It is apparent from this figure that a lot of statistically significant differences between C and MC cases in the content of all alpha helices (Fig. 6a), beta sheets (Fig. 6b), non-ordered structure components (Fig. 6d) as well as in the total area of the Amide I region (Fig. 6e) were found. As regards the content of beta turns (cf. Fig. 6c), six differences were not statistically significant. In particular, it was noted that the content of beta turns increased in SN, NAC and STR_E (of the left hemisphere). In Fig. 6d, we can see that the content of random coils in VTA, SN, STR_E and STR_F, as well as in the left side of both DMV and NAC, was markedly higher in the MC group compared with the control animals. Apart from that, as shown in Fig. 6a, the content of alpha helices was found to be significantly reduced for MC cases in VTA (*p* ≪ 0.05 for both hemispheres) and SN (*p* ≪ 0.05 for both hemispheres), as well as in the left side of both STR_E (*p* < 0.05) and STR_F (*p* = 0.032). As shown in Fig. 6b, the stimulated animals had an increased content of beta sheets in both VTA and CX. Moreover, for the MC group, the left side of STR_E as well as the right side of DMV was also enriched in beta sheets, though for the left side of both NAC and SN as well as for the right side of CX, the increase was not statistically significant. Last but not least, we see in Fig. 6e that the total area of the Amide I region for MC cases in DMV (*p* ≪ 0.05), VTA of the right hemisphere (*p* < 0.05), SN of the right hemisphere and NAC of the left hemisphere (*p* ≪ 0.05) was found to be significantly reduced except for both the left side of STR_F (*p* ≪ 0.05) and the right side of CX (*p* ≪ 0.05) where an increase occurred.

**Fig. 7:**
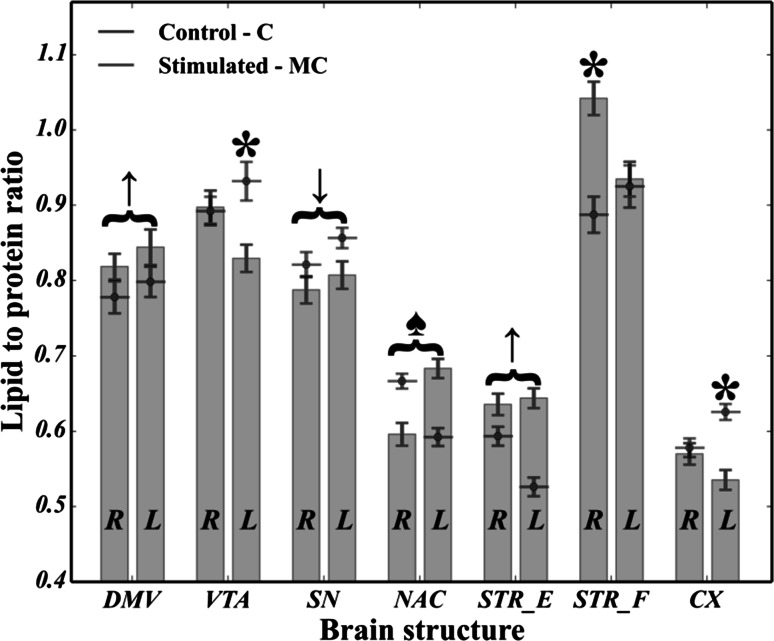
Comparison of changes in the lipid-to-protein ratio between the microchip-stimulated group (MC) and the control (C). R—right hemisphere; L—left hemisphere

As regards the lipid-to-protein ratio, it can be seen in Fig. 7 that all brain structures belonging to the dopaminergic system of the rats (except for some of the right hemisphere, namely VTA, CX and STR_F of the left hemisphere) were found to have strongly statistically significant differences (*p* ≪ 0.05) between the MC and C cases. Hence, it was noted that electrical stimulation affects the global level of lipids and proteins, and their ratio seems to be affected as well. It should be emphasized that the left side of both the mesocortical (VTA-CX) and part of nigrostriatal (SN-STR) dopaminergic pathways was found to be decreased (*p* ≪ 0.05) in the lipid-to-protein ratio, while for the DMV, STR_E and NAC of the left hemisphere, a significant (*p* ≪ 0.05) increase was noted.

**Fig. 8:**
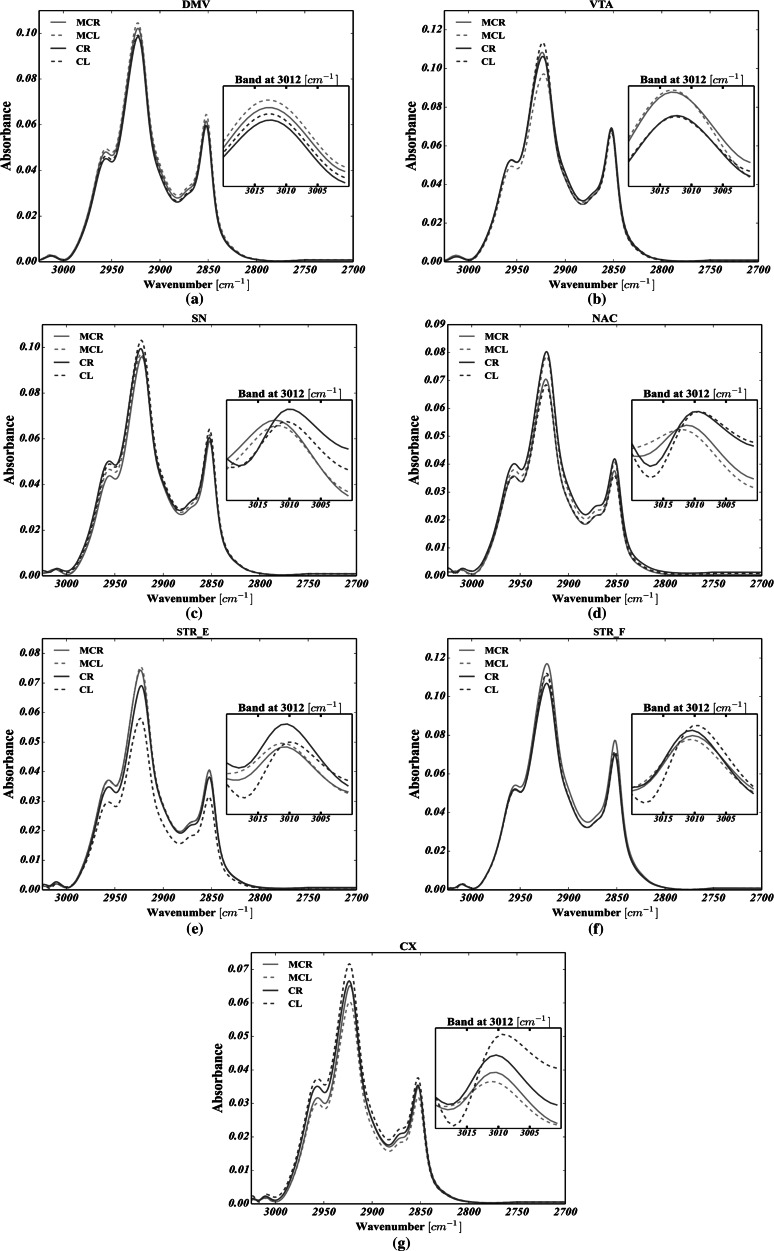
Global spectral changes in lipid massive for: **a** the dorsal motor nucleus of the vagus nerve (DMV); **b** the ventral tegmental area (VTA); **c** the substantia nigra SN; **d** the nucleus accumbens (NAC); **e** the corpus striatum spaces (STR_E); **f** the fiber bundles of striatum (STR_F); **g** the motor cortex (CX). CR—control group, right hemisphere; CL—control group, left hemisphere; MCR—microchip-stimulated group, right hemisphere; MCL—microchip-stimulated group, left hemisphere

**Fig. 9:**
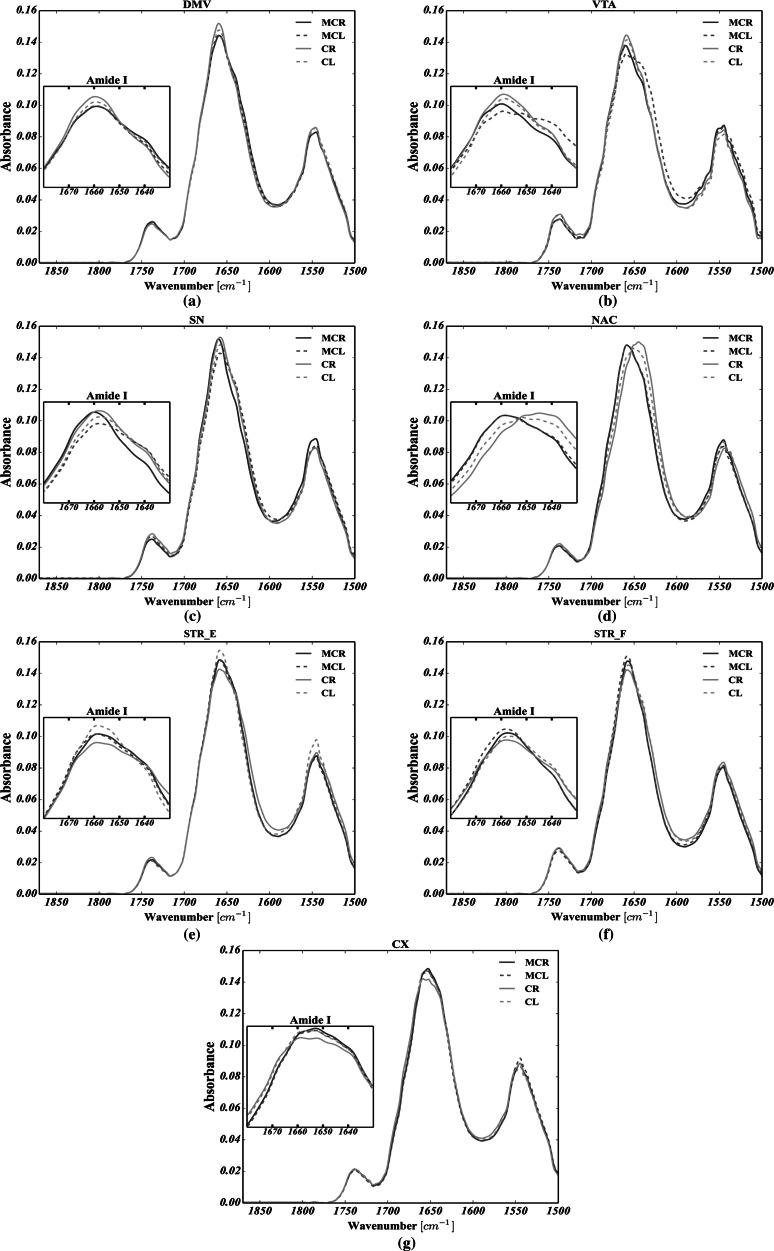
Global spectral changes in Amide I for: **a** dorsal motor nucleus of the vagus nerve (DMV); **b** ventral tegmental area (VTA); **c** substantia nigra (SN); **d** nucleus accumbens (NAC); **e** corpus striatum spaces (STR_E); **f** fiber bundles of striatum (STR_F); **g** motor cortex (CX). CR—control group, right hemisphere; CL—control group, left hemisphere; MCR—microchip-stimulated group, right hemisphere; MCL—microchip-stimulated group, left hemisphere

Average FTIRMS spectra, shown in Figs. 8 and 9 for C–H str. and Amide I–II spectral range, respectively, were calculated for each structure analyzed, based on all the spectra taken into account. By analyzing Fig. 8, it is apparent that the secondary structure of proteins was markedly changed in both VTA and SN of the left hemisphere upon electric stimulation, as their Amide I for MC group was markedly broadened, decreased and shifted toward lower wavenumbers. The most striking observation which emerges from the data shown in Fig. 9 is the shift of the olefinic band (3012 cm^−1^) toward higher wavenumbers for all brain structures analyzed in MC group compared with control. Noteworthy, in MC group, the mean FTIRMS profile of VTA of the left hemisphere revealed a dramatic decrease in the intensity of vibration modes arising from of C–H str.

## Discussion

In this study, the use of FTIRMS spectral imaging sets out with the aim of assessing any potential biomarkers for pathologies within the dopaminergic system of rats following peripheral vagus nerve stimulation. To begin with, from the curve fitting of the spectral range related to lipid functional groups (3020–2800 cm^−1^), we found that in virtually all the brain areas taken into account, both the FACLs (formula 1.2) and the level of lipid unsaturation (formula 1.1) were significantly affected in the MC group compared with the control. In short, by comparing Fig. 5a–c, it seems plausible that due to the occurrence of striking differences between C and MC, cases in both lipid saturation and unsaturation in case of VTA (both hemispheres), CX (particularly the left hemisphere) and NAC (particularly the left hemisphere), as parts of the mesocortical (VTA-CX) and mesolimbic (VTA-NAC) dopaminergic pathways (especially the left branch), might be particularly prone to lipid-related effects of vagus nerve stimulation. On top of that, our results provide compelling evidence that at the beginning of the dopaminergic system (SN, VTA), lipid composition would seem to be particularly affected by CVNS. It is conceivable that the right side of the mesolimbic pathway of the MC animals (VTA and NAC), due to elevated level of the *ν* = (CH)/*ν*_as_(CH_3_) ratio, is likely to be particularly vulnerable to changes in the level of fatty acid unsaturation. Significantly, it may be concluded that the whole nigrostriatal pathway (SN-STR) was markedly affected in terms of the total area integrations arising from C–H modes of vibrations. In turn, the VTA of the left hemisphere, which the mesolimbic and mesocortical pathways have in common, was found to have increased ratios of the *ν* = (CH)/*ν*_as_(CH_3_) or *ν*_as_(CH_2_)/*ν*_as_(CH_3_). A plausible explanation for these results may be an increase in both lipid unsaturation and/or saturation in the VTA, which could be due to the enhanced synthesis of phospholipids, suggesting that CVNS might have enhanced lipogenesis within the VTA. Another important finding was that the right branches of both the nigrostriatal and mesocortical dopaminergic pathways were found to be markedly susceptible to the lipid-related effects of CVNS. In particular, it was found that an increase in the ν = (CH)/ν_as_(CH_3_) ratio coincided with an increase in the ν_as_(CH_2_)/ν_as_(CH_3_) ratio in both SN and CX. The reason for this may have something to do with the increased expression of n-3 and n-6 polyunsaturated fatty acids (PUFA). It has been reported that PUFA were particularly liable to the effects of oxidative stress due to their unsaturated chemical bonds (C=C). Hence, it is likely that this phenomenon might predispose the mesolimbic and nigrostriatal pathways to subsequent lipid peroxidation, and thus, it could underlie the development of in their early stages pathologies within the dopaminergic system of rats (Ruipérez et al. 2010). Interestingly, the STR of the right side had an even more pronounced lipid-related response to stimulation; the ν = (CH)/ν_as_(CH_3_) ratio was found to be markedly reduced in stimulated animals, whereas the ν_as_(CH_2_)/ν_as_(CH_3_) ratio was increased in the MC group. A possible explanation for this might be that lipids within the STR were less unsaturated. This, therefore, may shed light on lipid degradation in the STR as a potential consequence of vagal nerve stimulation in rats. This view is supported by Abdel-Salam et al. (2013), who pointed out that the mechanisms which are induced upon damage to the vagus nerve may lead to complex lipid-related alterations within the dopaminergic system, so that subdiaphragmatic vagotomy (reported in our previous studies by Ziomber et al. (2012)) as well as capsaicin-induced dysfunction of the capsaicin-sensitive vagal afferent fibers increased lipid peroxidation via oxidative stress (Ziomber et al. 2012). It is likely, therefore, that the integrity of the vagus nerve may be crucial in maintaining the redox status during basal conditions in the dopaminergic system of rats (Abdel-Salam et al. 2013). Compatibly, recent evidence indicates that an increased inflammatory response, along with an elevated concentration of Fe, promotes the Fenton reaction, leading to lipid peroxidation and degradation via oxidative stress (Hwang 2013; Yoritaka et al. 1996). Thereby, the findings of the current study seem to be in line with those of Szczerbowska-Boruchowska et al. (2012), who reported that the level of selected elements in the STR, including, among others iron, was found to be markedly increased in the MC group (Zaleska et al. 1989; Szczerbowska-Boruchowska et al. 2012). Our findings, therefore, may support previous research into this brain area, linking the lipid-related effects of oxidative stress with an increased accumulation of iron. One can notice that lipid-related alterations in the origins of both the mesocortical and the nigrostriatal DA pathways were markedly different from those reported in their terminal fields. Hence, it could conceivably be hypothesized that although the lipid-related changes in CVNS might be conditioned upon localization, it seems possible that all of them might arise as a result of the complex biochemical effects of oxidative stress. This conclusion also accords with our observations that the C=C (olefinic) band centered around 3010 cm^−1^ was found to be more or less shifted toward higher frequencies in all dopamine-related structures for the MC group, and so it is likely that the chemical surrounding of unsaturated fatty acids within dopaminergic system of rats could be significantly changed following CVNS (see Fig. 8).

As mentioned, apart from lipid-related changes, it was found that CVNS influenced the protein composition as well. The results presented in Fig. 6a–e show that the VTA, NAC, SN, STR_E, STR_F and CX, as the parts of the mesolimbic (VTA-NAC), mesocortical (VTA-CX) and nigrostriatal (SN-STR) dopaminergic pathways, are particularly affected by vagus nerve stimulation due to the co-occurrence of statistically significant changes in the content of all non-ordered structure components, alpha helices, beta sheets and the total area of the Amide I. The left branch of the nigrostriatal pathway especially seems to be affected more than the right branch. So, it could be inferred that a decrease in the content of alpha helices and an increase in both the content of random coils and beta structures (sheets and turns), along with a severe decrease in the total area of Amide I, are likely to represent the most striking protein-related effects of vagus nerve stimulation in rats. In turn, based on Amide I (1800–1500 cm^−1^) band decomposition, a decrease in the integrated intensity of Amide I was usually accompanied by a decrease in the content of alpha helices, together with an elevated number of either beta structures or random coils. At the same time, virtually the whole left branch of the dopaminergic system showed a marked increase in the content of random coils. Most notably, SN and VTA, which represent the origin of dopamine efferent fibers, were affected in a largely similar fashion to that described above. Interestingly, STR had the same adverse biochemical effect as SN, suggesting that the whole nigrostriatal pathway was changed upon electrical stimulation to a similar extent. Hence, since the integrated intensity of the Amide I band, which is known to indicate the content of proteins in the tissue, was found to be significantly decreased, it could be hypothesized that protein degradation was involved in the pathological changes related to CVNS in rats. It thus became apparent that a decrease in the number of alpha helices with a correlated increase in the number of both beta forms and random coils may be associated with the degradation of correctly folded proteins and the generation of misfolded ones (Turker et al. 2014). This comes as no surprise and might be explained in the light of the pathomechanisms that underlie PD (Ruipérez et al. 2010; Hwang 2013; Seet et al. 2010). Thus, to date, it has been demonstrated that proteolytic stress, which is responsible for the presynaptic accumulation of ubiquitinated, misfolded, aggregated and oxidized deposits of alpha synuclein in SN and STR, underlies the initial changes associated with the inevitable development of PD (Irwin et al. 2013; Yoritaka et al. 1996). It is, therefore, clear that the neurodegeneration process may start at the nerve terminals (SN) of dopaminergic neurons, further expanding to the other parts of the whole dopaminergic system via what is known as retrograde dopaminergic neuron degeneration (Morales et al. 2013). Hence, it is apparent that our study produced results which corroborate those findings, because as the strong shifts in the protein secondary structure occurred for SN, it could be inferred that CVNS affected the origin of dopamine efferent fibers in a manner that was similar to the initial course of the preclinical PD. Given the protein-related changes in VTA, one may conclude that the onset of the mesolimbic reward system, which is considered to play a crucial role in the control of food intake, showed even more intense changes in protein secondary structure than those observed in SN (Kawahara et al. 2013). Hence, it is likely that this could be due to the direct exposition of VTA on incoming projections from DMV. Interestingly, the mean spectral profile attributed to VTA of the left hemisphere was found to be markedly decreased at 1654 cm^−1^ and strongly broadened toward lower frequencies for the MC group (see Fig. 9), suggesting a strong contribution of alpha helices degradation and the generation of misfolded peptides (beta forms and random coils) in this group, though its area of Amide I remained unchanged. Indeed, based on these three observations, our results confirm that it is possible to hypothesize that the irreversible transition between alpha helices and misfolded proteins might be a result of the chronic stimulation of the vagus nerve in VTA.

## Conclusions

This paper has examined the impact of CVNS on the biochemical composition of the dopaminergic system of rats. Returning to the hypothesis posed at the beginning of this study, it is now possible to state that vagus nerve stimulation induced lipid- and protein- related changes within various dopamine-related structures in the rat brain. This confirms our preliminary hypothesis that the activity of the vagus nerve may influence the whole dopaminergic system, suggesting a role for gastrointestinal system during the development of dopamine-related disorders. In particular, this study has shown that the left hemisphere, ipsilateral to the electric stimulation, appeared to undergo many lipid- and protein-related biochemical changes, though there was a weaker protein-related effect on the contralateral (right) side in the MC group. In turn, because the DMV, considered a point of departure for early changes during the progression of PD, was found to be predisposal to the effects of oxidative stress neurotoxicity, it may be concluded that macromolecular changes in brain structures caused by a dysfunction of the vagus nerve may reflect initial pathological processes within the dopaminergic system of rats. On top of that, since our previous research has proved that vagus nerve stimulation produced a significant inhibition of dopamine signaling in a similar way to physiological vagotomy, it may be inferred from the present study that both the physiology and biomolecular composition of all the dopamine-related brain regions analyzed were markedly affected. In short, the study has gone some way toward enhancing our understanding of the “physiological link” between the gastrointestinal system and the central nervous system. Our investigations may provide feasible additional explanations for the variety of psychiatric symptoms in patients with gastrointestinal disorders such as inflammatory bowel disease (Zhang et al. 2013), or irritable bowel syndrome (Orzechowska et al. 2013), and shed some light on the role of the vagus nerve in the early stages of either neurological (i.e., PD) or psychiatric (i.e., depressive) dopamine-related disorders.

## Abbreviations

PD: Parkinson’s disease
NAC: Nucleus accumbens
SN: Substantia nigra
STR: Striatum
STR_F: Striatum fiber bundles
STR_E: Corpus striatum spaces
DMV: Dorsal motor nucleus of the vagus nerve
FTIRMS: Fourier transform infrared microspectroscopy
CVNS: The chronic stimulation of the vagal nerve
UNSAT: The level of lipid unsaturation
FACL: The fatty acyl chain length
IR: Infrared

## References

[CR1] Abbott RD, Petrovitch H, White LR, Masaki KH, Tanner CM, Curb JD, Grandinetti A, Blanchette PL, Popper JS, Ross GW (2001). Frequency of bowel movements and the future risk of Parkinson’s disease. Neurology.

[CR2] Abdel-Salam OM, Abdel-Rahman RF, Sleem AA, Mosry FA, Sharaf HA (2013). Effects of afferent and efferent denervation of vagal nerve on endotoxin-induced oxidative stress in rats. Journal of Neural Transmission.

[CR3] Alhadeff AL, Rupprecht LE, Hayes MR (2012). GLP-1 neurons in the nucleus of the solitary tract project directly to the ventral tegmental area and nucleus accumbens to control for food intake. Endocrinology.

[CR4] Braak H, Del Tredici K, Rüb U, De Vos RAI, Ernst NH, Steur J, Braak E (2003). Staging of brain pathology related to sporadic Parkinson’s disease. Neurobiology of Aging.

[CR5] de Lau LM, Breteler MM (2006). Lancet Neurology.

[CR6] Dickson DW (2012). Parkinson’s disease and parkinsonism: neuropathology. Cold Spring Harbor perspectives in medicine.

[CR7] Fabelo N, Martín V, Marín R, Moreno D, Ferrer I, Díaz M (2014). Altered lipid composition in cortical lipid rafts occurs at early stages of sporadic Alzheimer’s disease and facilitates APP/BACE1 interactions. Neurobiology of Aging.

[CR8] Fukunishi I, Hosokawa K, Ozaki S (1991). Depression antedating the onset of Parkinson’s disease. Psychiatry and Clinical Neurosciences.

[CR9] Gough KM, Tzadub L, Kastyak MZ, Kuzyk AC, Julian RJ (2010). Theoretical and experimental considerations for interpretation of Amide I bands in tissue. Vibrational Spectroscopy.

[CR10] Greene JG (2014). Causes and consequences of degeneration of the dorsal motor nucleus of the vagus nerve in Parkinson’s disease. Antioxidants and Redox Signaling.

[CR11] Guze BH, Barrio JC (1991). The etiology of depression in Parkinson’s disease patients. Psychosomatics.

[CR12] Hunter JD (2007). Matplotlib: A 2D graphics environment. Computing In Science and Engineering.

[CR13] Hwang O (2013). Role of oxidative stress in Parkinson’s disease. Experimental Neurobiology.

[CR14] Irwin DJ, Lee VMY, Trojanowski JQ (2013). Parkinson’s disease dementia: convergence of a-synuclein, tau and amyloid-b pathologies. Nature Reviews Neuroscience.

[CR15] Kawahara Y, Kaneko F, Yamada M, Kishikawa Y, Kawahara H, Nishi A (2013). Food reward-sensitive interaction of ghrelin and opioid receptor pathways in mesolimbic dopamine system. Neuropharmacology.

[CR16] Kolta Z, Debreczeny M, Szalonta B (1999). Separable contributions of ordered and disordered lipid fatty Acyl chain segments to nCH2 bands in model and biological membranes: a Fourier transform infrared spectroscopic study. Biospectroscopy.

[CR17] Kostić VS, Lesić D, Doder M, Marinković J, Filipowić S (1996). Prolactin and cortisol responses to fenfluramine in Parkinson’s disease. Biological Psychiatry.

[CR35] Leskovjan C, Kretlow A, Miller LM (2010). Fourier transform infrared imaging showing reduced unsaturated lipid content in the hippocampus of a mouse model of Alzheimer’s disease. Analytical chemistry..

[CR18] Mellers JD, Quinn NP, Ron MA (1995). Psychotic and depressive symptoms in Parkinson’s disease. A study of the growth hormone response to apomorphine. British Journal of Psychiatry.

[CR19] Merchenthaler I, Lane M, Shughrue P (1999). Distribution of pre-pro-glucagon and glucagon-like peptide-1 receptor messenger RNAs in the rat central nervous system. Journal of Comparative Neurology.

[CR20] Morales I, Sabate M, Rodriguez M (2013). Striatal glutamate induces retrograde excitotoxicity and neuronal degeneration of intralaminar thalamic nuclei: their potential relevance for Parkinson’s disease. European Journal of Neuroscience.

[CR21] Orzechowska A, Talarowska M, Zboralski K, Florkowski A, Gałecki P (2013). Subjective evaluation of symptoms and effects of treatment and the intensity of the stress and anxiety levels among patients with selected diseases of the skin and gastrointestinaltract. Psychiatria Polska.

[CR22] Petibois C, Déléris G (2006). Chemical mapping of tumor progression by FTIR imaging: towards molecular histopathology. Trends in Biotechnology.

[CR23] Rinaman L (2010). Ascending projections from the caudal visceral nucleus of the solitary tract to brain regions involved in food intake and energy expenditure. Brain Research.

[CR24] Ronald F, Pfeiffer RF, Wszolek ZK, Ebadi M (2012). Parkinson’s disease.

[CR25] Ruipérez V, Darios F, Davletov B (2010). Alpha-synuclein, lipids and Parkinson’s disease. Progress in Lipid Research.

[CR26] Seet RC, Lee CY, Lim EC, Tan JJ, Quek AM, Chong WL, Looi WF, Huang SH, Wang H, Chan YH, Halliwell B (2010). Oxidative damage in Parkinson disease: Measurement using accurate biomarkers. Free Radical Biology and Medicine.

[CR27] Stuart BH (2004). Infrared spectroscopy: Fundamentals and Applications.

[CR28] Surowka AD, Adamek D, Radwanska E, Szczerbowska-Boruchowska M (2014). Variability of protein and lipid composition of human substantia nigra in aging: Fourier transform infrared microspectroscopy study. Neurochemistry International.

[CR29] Szczerbowska-Boruchowska M, Krygowska-Wajs A, Ziomber A, Thor P, Wrobel P, Bukowczan M, Zizak I (2012). The influence of electrical stimulation of vagus nerve on elemental composition of dopamine related brain structures in rats. Neurochemistry International.

[CR30] Turker S, Ilbay G, Severcan M, Severcan F (2014). Investigation of compositional, structural, and dynamical changes of pentylenetetrazol-induced seizures on a rat brain by FT-IR spectroscopy. Analytical Chemistry.

[CR31] van der Walt S, Colbert SC, Varoquaux G (2011). The NumPy array: A structure for efficient numerical computation. Computing in Science and Engineering.

[CR32] Wang ZY, Lian H, Cai QQ, Song HY, Zhang XL, Zhou L (2014). No direct projection is observed from the substantia nigra to the dorsal vagus complex in the rat. J Parkinsons Dis..

[CR33] Wehbe K, Pineau R, Eimer S, Vital A, Loiseau H, Déléris G (2010). Differentiation between normal and tumor vasculature of animal and human glioma by FTIR imaging. Analyst.

[CR34] Wolfgang H (2010). Gastrointestinal dysfunction in Parkinson’s disease. Journal of the Neurological Sciences.

[CR36] Yoritaka A, Hattori N, Uchida K, Tanaka M, Stadtman ER, Mizuno Y (1996). Immunohistochemical detection of 4-hydroxynonenal protein adducts in Parkinson disease. Proceedings of the National Academy of Sciences of USA.

[CR37] Zaleska MM, Nagy K, Floyd RA (1989). Iron-induced lipid peroxidation and inhibition of dopamine synthesis in striatum synaptosomes. Neurochemical Research.

[CR38] Zhang CK, Hewett J, Hemming J, Grant T, Zhao H, Abraham C, Oikonomou I, Kanakia M, Cho JH, Proctor DD (2013). The influence of depression on quality of life in patients with inflammatory bowel disease. Inflammatory Bowel Diseases.

[CR39] Ziomber A, Thor P, Krygowska-Wajs A, Zalecki T, Moskala M, Romanska I, Michaluk J, Antkiewicz-Michaluk L (2012). Chronic stimulation of the vagus nerve function leads to inhibition of dopamine but not serotonin neurons in rat brain structures. Pharmacological Reports.

